# Neural network based estimates of the climate impact on mortality in Germany: application to storyline climate simulations

**DOI:** 10.1038/s41598-024-77398-3

**Published:** 2024-10-30

**Authors:** R. Schachtschneider, J. Saynisch-Wagner, A. Sánchez-Benítez, M. Thomas

**Affiliations:** 1 Helmholtz Centre Potsdam GFZ German Research Centre for Geosciences, Telegrafenberg, 14473 Potsdam, Germany; 2 Alfred Wegener Institute, Helmholtz Centre for Polar and Marine Research, Am Handelshafen 12, 27570 Bremerhaven, Germany; 3 Free University Berlin, Kaiserswerther Str. 16 - 18, 14195 Berlin, Germany

**Keywords:** Climate change, Machine learning, Mortality prediction, Human health, Storyline simulations, Climate-change impacts, Risk factors

## Abstract

The aim of this work is the prediction of heat-related mortality for Germany under future, i.e. hotter, climate conditions. The prediction is made based on 2m temperature data from climate storyline simulations using machine learning techniques. We use an echo state network for linking the outputs of storyline climate simulations to the target data. The target data are all-cause mortality rates of Germany for all ages. The network is trained with present day climate model outputs. Model outputs of future, i.e. 2K and 4K warmer, storylines are used to predict mortality rates under such climatic conditions. We find that we can train an echo state network with recent temperature data and mortality and make plausible predictions about expected developments of mortality in Germany based on future climate storylines. The trained network can successfully predict mortality rates for future climate conditions. We find increased mortality during the summer months which is attributed to the presence of more severe heat waves. The mortality decrease found during winter can be explained milder winters leading to fewer deaths caused by respiratory diseases. However, mortality in winter is largely influenced by other factors such as influenza waves or vaccination rate and explainability due to temperature is limited.

## Introduction

Machine learning (ML) has become a helpful tool in many areas of science and medicine^1,2^. It comprises algorithms for revealing non-linear relationships between input and output variables and can be helpful in many classification tasks or regression problems.

In medicine, ML can teach researchers new ways of studying diseases, producing medicines or treating patients and can be applied for early disease detection where severe conditions are likely to manifest at a later time^2–4^ used a gradient boosting model to predict the likelihood of acute myocardial infarction for patients.^5^ trained a deep neural network for classification of skin lesions into skin cancer risk categories.

Mortality prediction has been conducted in various fields of medicine and for different diseases such as Chronic Obstructive Pulmonary Disease^6^, COVID-19^7^, neonatal mortality^8^, or general mortality risk in populations with coronary artery disease^9^ or general elderly populations^10^. Taylor et al.^11^ predict mortality of hospitalized patients with sepsis in a data-driven ML-approach. The impact of climate change on mortality due to various diseases have been studied with the help of ML, e.g. for Malaria^12^, myocardial infarctions^13^, or chronic obstructive pulmonary disease^6^.

Impacts of climate change and increasing numbers of death from heat disorder have been studied, e.g. by^14–17^. Lee et al.^18^ investigated the influence of heat exposure on mental health and connected implications following climate change. This includes mortality due to suicides, mental disorders, and violence among others. Hirano et al.^19^ developed a ML-based model for mortality prediction for heat-related illnesses on the base of data from hospitalized patients in Japan. Kim & Kim^20^ used a random forest model for heat-related mortality prediction in a detailed area within a city with various climatic, demographic and socio-economic sectors. Winklmayr et al.^21^ employ a generalized additive model for the estimation of heat-related deaths in Germany for the period 1992–2021. They can characterize long-term trends and quantify the effect of heat on mortality over the years. Mistry et al.^22^ compare results of mortality models based on temperature measurement data at a number of weather stations to models based on global reanalyses of weather observations and climate models by the European Centre for Medium-Range Weather Forecasts (ECMWF). They find that reanalysis data are a valid alternative source in temperature-related health risk studies.

In this study we predict the mortality rate in Germany for two different climate storylines computed in the project SCENIC using an echo state network (ESN). We show that the network can be trained on five years of monthly maximum temperature input data and monthly mortality rates for Germany as target data. With the trained network we can reliably predict mortality rates in Germany for the two given future climate storylines.

## Method

### Background

Artificial neural networks (ANN) are inspired by biological examples^23^. Neurons in the brain are interconnected and capable of fulfilling enormous cognitive tasks with utmost precision. An ANN consists of a large number of artificial neurons that mimic the behavior of connected neurons in the brain. The artificial neurons have an activation threshold above which they pass on information and weights that encode the importance of the neuron connection. Several neurons are organized in a layer and many layers make up a neural network. The input layer receives information from the outside world, a number of hidden layers further processes the information from the input layer, and the output layer transforms the information from the network into a result. The shape of the result depends on the purpose the network is designed for. There are numerous kinds of layered networks for purposes of , e.g., classification, regression, clustering or object detection.

In the training phase the weights of an ANN are adjusted. In supervised learning this is achieved by providing input samples with the corresponding known output. Through iterative back-propagation techniques the weights of the ANN nodes are adjusted to minimize the misfit between the network output and the known result. The challenge is to avoid over-fitting and obtain an ANN that performs well on unknown data. Unsupervised learning uses only input data to learn the distribution of the input variables. It is useful for clustering, compression, or feature extraction^23^.

A recurrent neural network (RNN) is a network with cyclic connections in which the neurons can send feedback signals to each other^24^. They are more powerful than acyclic feed forward neural networks (FNNs) in that they can create and process memories of arbitrary sequences of input patterns^25^. Therefore, while FNNs represent functions, RNNs represent dynamical systems which may develop a self-sustained temporal activation even in the absence of input^26^. Supervised Long Short-Term Memory (LSTM) RNNs can discover and memorize events that happened thousands of time steps ago^25^. Training of RNNs, however, is challenging. The well-known feed-forward back-propagation algorithm cannot be directly used because of the cyclic nature of the reservoir. One simple solution to overcome this difficulty is to neglect all indirect pathway (i.e. such containing cycles). However, this is a rather coarse approximation of the optimization problem^27^. There exist two basic ways for calculating the exact gradient fo the output with respect to the weights: forward and backward methods^27^. The forward method solves a linear dynamical equation system for the effect of a small change in a weight on the network state trajectory. While this can be calculated concurrently with the network dynamics the computational costs are quite high. In the backward method the causes of the output error are computed backward in time. In case of discrete time steps this can be achieved by setting up a multi-layer feed-forward network for unrolling the multi-step evolution of the network. More comprehensive overviews over RNNs and training algorithms are given by^26–29^.

### The echo state network

**Fig. 1:**
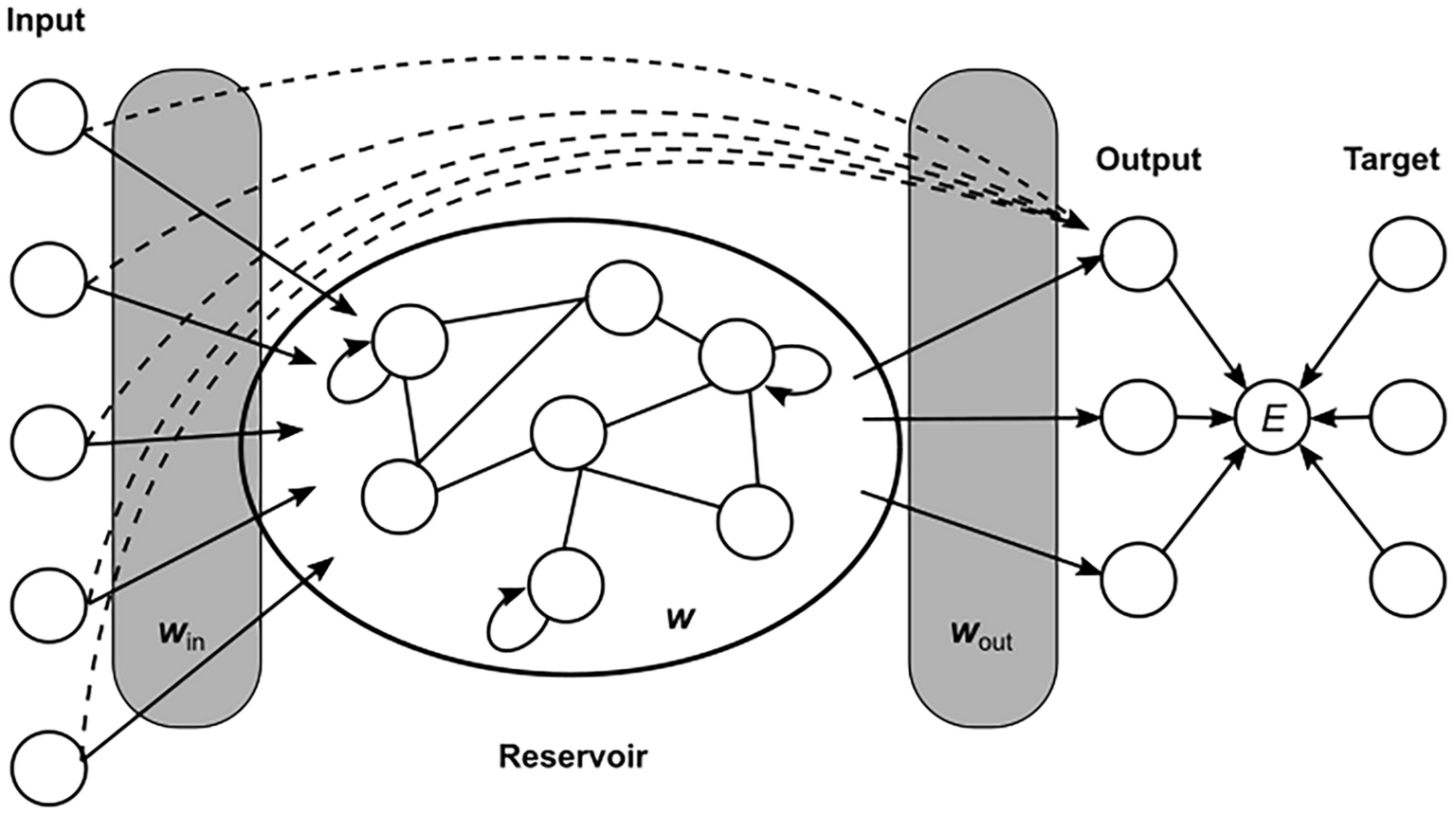
Schematic of an echo state network. The input layer is connected to a reservoir. In the reservoir there are connections between some of the nodes, including recurrent connections. The output layer is connected to the reservoir and to the input layer. After adaption of the neuron activations in the reservoir, the output weights are determined by a regression that minimizes the misfit, *E*, between prediction and target.

The ESN is a special form of a recurrent neural network that was introduced independently by^30,31^. Originally, it incorporates an algorithm for supervised learning in which only the output layer is modified^27^. A schematic of an ESN is shown in Fig. 1. ESNs consist of an input layer, a reservoir, and an output layer. The output layer is connected to the reservoir and the input layer. The reservoir contains cyclic and recurrent connections. It should be large, sparse and randomly connected^26^ . This allows the network to process information from earlier inputs than the current step since input information can cycle in the reservoir and influence later steps as well. Another important property is the so-called echo state. This means that the effect of input data vanishes gradually over time. This way an ESN can simulate a dynamic system and learn complex and time dependent connections between input data and target values. In this study it learns the connection between temperature distributions and mortality.

Our network realization is based on the description in^32^. The input weights are initialized randomly from a uniform distribution over the interval $[-0.5, 0.5]$. The reservoir weights are initialized by the same procedure and in addition a fraction $(1-c)$ of the weights, where $c \in [0, 1]$ is the connectivity of the reservoir, is set to zero. During the training process, the reservoir weights remain unchanged. Only the neuron activations are updated based on training input. The neuron activation determines whether a neuron is important and will be activated or not. The activation values are updated using

1 $$\begin{aligned} \textbf{x}_{n} = (1-\alpha ) \textbf{x}_{n-1} + \alpha \tanh (\textbf{W}_{\textrm{in}}[1;\textbf{u}_n] + \textbf{W}\textbf{x}_{n-1}) \end{aligned}$$

where $\textbf{x}_{n}$ are the neuron activations at step *n*, $\textbf{W}_{\textrm{in}}$ is the input weight matrix, $\textbf{u}$ is a vector containing the input data at step *n*, $[\cdot ;\cdot ]$ denotes a vertical vector concatenation, $\textbf{W}$ is the recurrent weight matrix of the reservoir, and $\alpha \in (0, 1]$ is the leakage parameter. The leakage parameter determines the proportion of the neuron activation update due to new input data at step *n*. Finally, the output weight matrix is determined by a ridge regression that minimizes the misfit between outputs and targets in a least squares sense. The ESN is described in more detail by^32^. The ESN parameters used in this study are given in Table 1.

**Table 1:** Parameters of the echo state network used in this study. $\alpha$ is the leakage parameter, $r_{\text {s}}$ is the spectral radius of the reservoir weights matrix, *c* is the connectivity of the reservoir, and $\lambda$ is the regression coefficient in the ridge regression of the output weights matrix.

Parameter	Reservoir size	$\alpha$	$r_{\text {s}}$	*c*	$\lambda$
Value	9000	0.5	1.25	0.5	1e-8

The trained network can then be used to either forecast new values of the time series of input data from the known inputs in the past or to compute the response to new inputs. Here, we use the latter functionality since the goal is to predict socio-economic parameters for new given inputs (i.e. in warmer world scenarios). The output is then determined by

2 $$\begin{aligned} \textbf{y}_{n} = \textbf{W}_{\textrm{out}}[1;\textbf{u}_{n};\textbf{x}_{n}] \end{aligned}$$

where $\textbf{W}_{\textrm{out}}$ is the matrix of output weights, $[\cdot ;\cdot ;\cdot ]$ denotes the vertical concatenation of vectors, $\textbf{u}_{n}$ is the input vector, and $\textbf{x}_{n}$ is the vector of neuron activations. Thus, the output at a certain time step is determined by both, the state of the reservoir and directly by the input data at that time. As indicated in Fig. 1, an error measure is calculated from the predictions and the target values. We choose the root-mean-square (RMS) error.

As the network is initialized with random values, the network state after training can be different for each realization. Therefore, we compute an ensemble of trained ESNs and predictions and calculate the arithmetic mean from the ensemble of predictions. The ensemble size is 25. The statistics of the ensemble are used to estimate the robustness of the approach.

## Data

As input data for training the ESN we used outputs of global climate simulations. The used simulations are so-called climate storyline simulations. They recreate extreme weather events in climate models by keeping some boundary conditions (in this case winds in the higher troposphere) close to states of the atmosphere observed in the past during those events. This process is called nudging. When different climate conditions (e.g. elevated CO2 concentrations) are applied to the climate model with the same nudging, it is possible to study how the extreme event would enfold under those new conditions. The storyline simulations are computed using a model developed at the Alfred Wegener Institute, Helmholtz Centre for Polar and Marine Research (AWI). The model is called AWI CM1 and a description can be found in^33^. From the various outputs of the AWI CM1 model we use temperature fields at 2m above ground level as input data for the network.

For the current time scenario the tropospheric winds in the climate model are nudged to ECMWF weather reanalysis data (ERA5). The two additional future climate conditions considered are elevated concentrations of CO2 that result in increased global mean temperatures of +2K and +4K scenarios w.r.t. pre-industrial times, respectively. The method for computing climate storylines is described in detail in^34^.

**Fig. 2:**
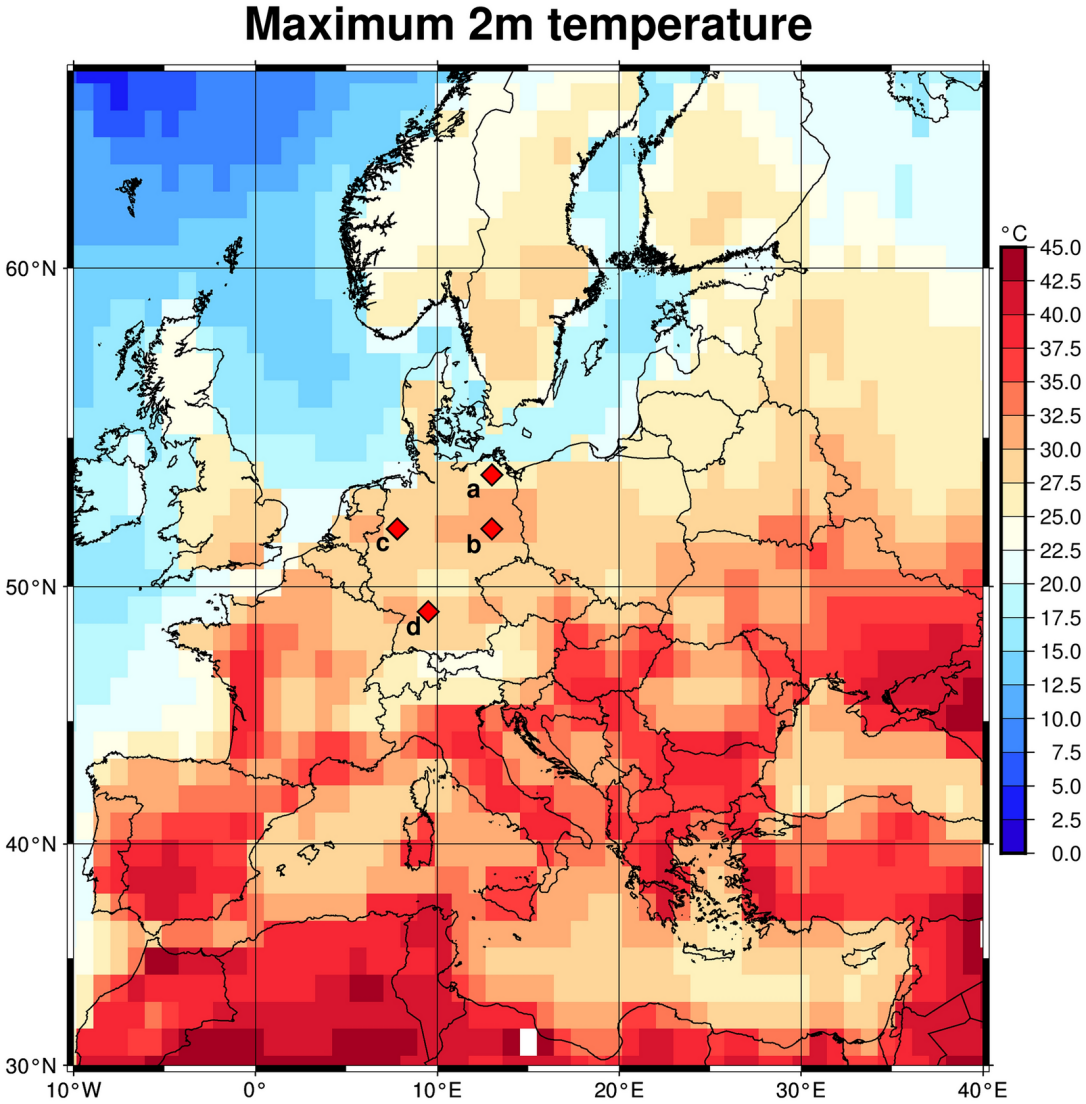
Example of temperature input data. Shown are the monthly maximal values at each grid point for July 2017. The input data consist of one 2D distribution of the 2m temperature for each input month. For the analysis, temperatures are normalized such that all values lie in the interval [0,1] over the whole investigation period. The red diamonds denote the locations of temperature time series shown in Fig. 3. This figure was produced with GMT 6.4.0^36^.

The climate storyline simulations that constitute our training data are available in a period from 2015 to 2021. The warmer world storylines that are used for the mortality rate predictions are available in a period from 2017 to 2021. In order to exclude excess mortality due to the COVID-19 pandemic we restrict the training and the prediction to before 2020. From the AWI CM1 model we used hourly 2m temperature fields from which we compute monthly maximum values. The data have a $1^{\circ }\times 1^{\circ }$ spatial resolution. We consider a region within the longitudinal range from $10^{\circ }$W to $40^{\circ }$E and the latitudinal range from $30^{\circ }$N to $65^{\circ }$N. This covers a region large enough to include all influences on people in Germany but reduces the computational effort compared to using the global data set. The temperatures are normalized such that the interval [$-13^{\circ }$C, $47^{\circ }$C] maps to [0,1]. The temperature data used as inputs in this study have been published by^35^. An example of a temperature field before normalization used as input is given in Fig. 2.

**Fig. 3:**
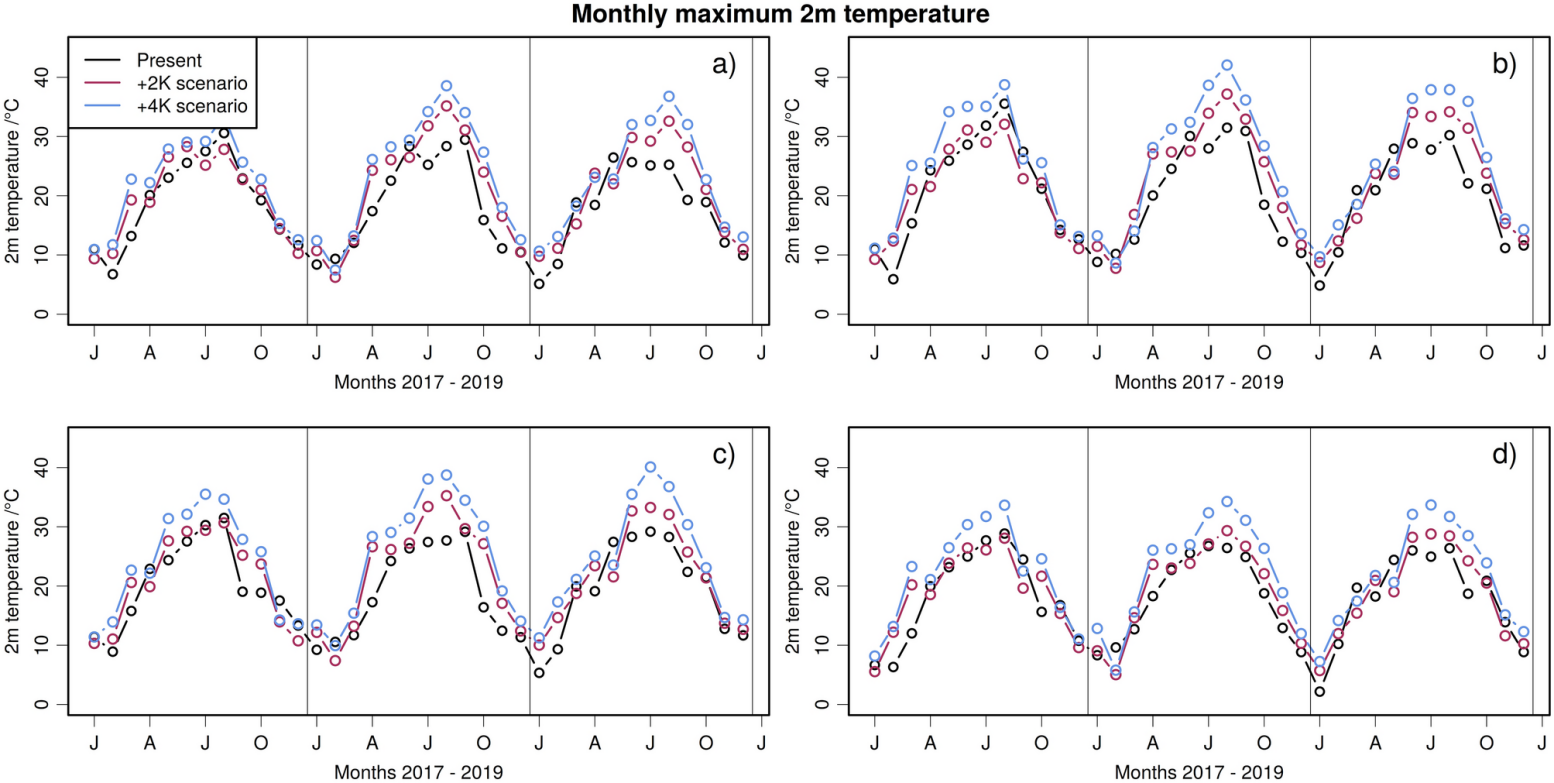
Examples of monthly maximum temperature development over time for all considered scenarios. The locations of the profiles are shown in Fig. 2. The temperature differences between current and future scenarios can be much larger than the average difference.

Figure 3 shows examples of temperature time series at four locations in Germany. Even though the global mean temperature is only 2K or 4K higher than in pre-industrial times, the local maximum temperature difference can be much higher.

The target data are monthly mortality values published by the German Federal Statistical Office. They are available under the code *12613-03-01-4-B* at https://www.regionalstatistik.de^37^. The data are all-cause mortality values over all ages. The mortality was scaled by the population of each federal state to yield the mortality per 100,000 inhabitants. The population statistics are available at the same website under the code *12411-05-01-4-B*. The population data have annual values so monthly values were obtained by linear interpolation where the annual values were assigned to the middle of each year.

## Results

**Fig. 4:**
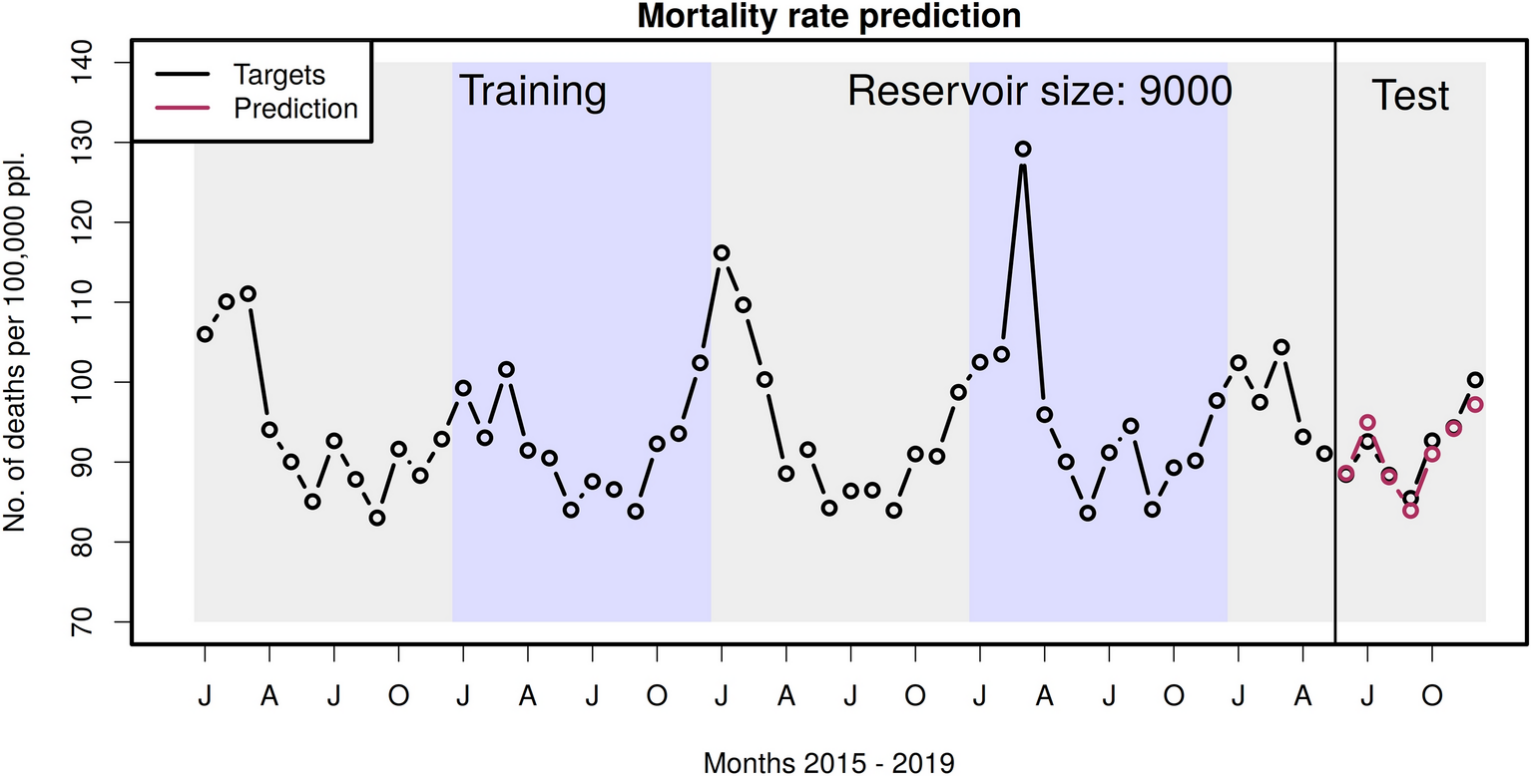
Training of the echo state network. This panel shows the five years of target data used for training and testing. The test period is seven months from June to December 2019 in the right part of the panel. The RMS error in the test period is 1.7, i.e. $\approx$ 1.9%.

With the described ESN we are able to predict mortality rates in Germany from monthly maps of maximum 2m temperatures in Europe. Figure 4 shows the time series of the target values used for training the network (left part) and the subsequent test predictions (right part, red circles). In the test months, the prediction of mortality is very accurate given the short training period. The RMS error is 1.7, i.e. $\approx$ 1.9% in the test period.

**Fig. 5:**
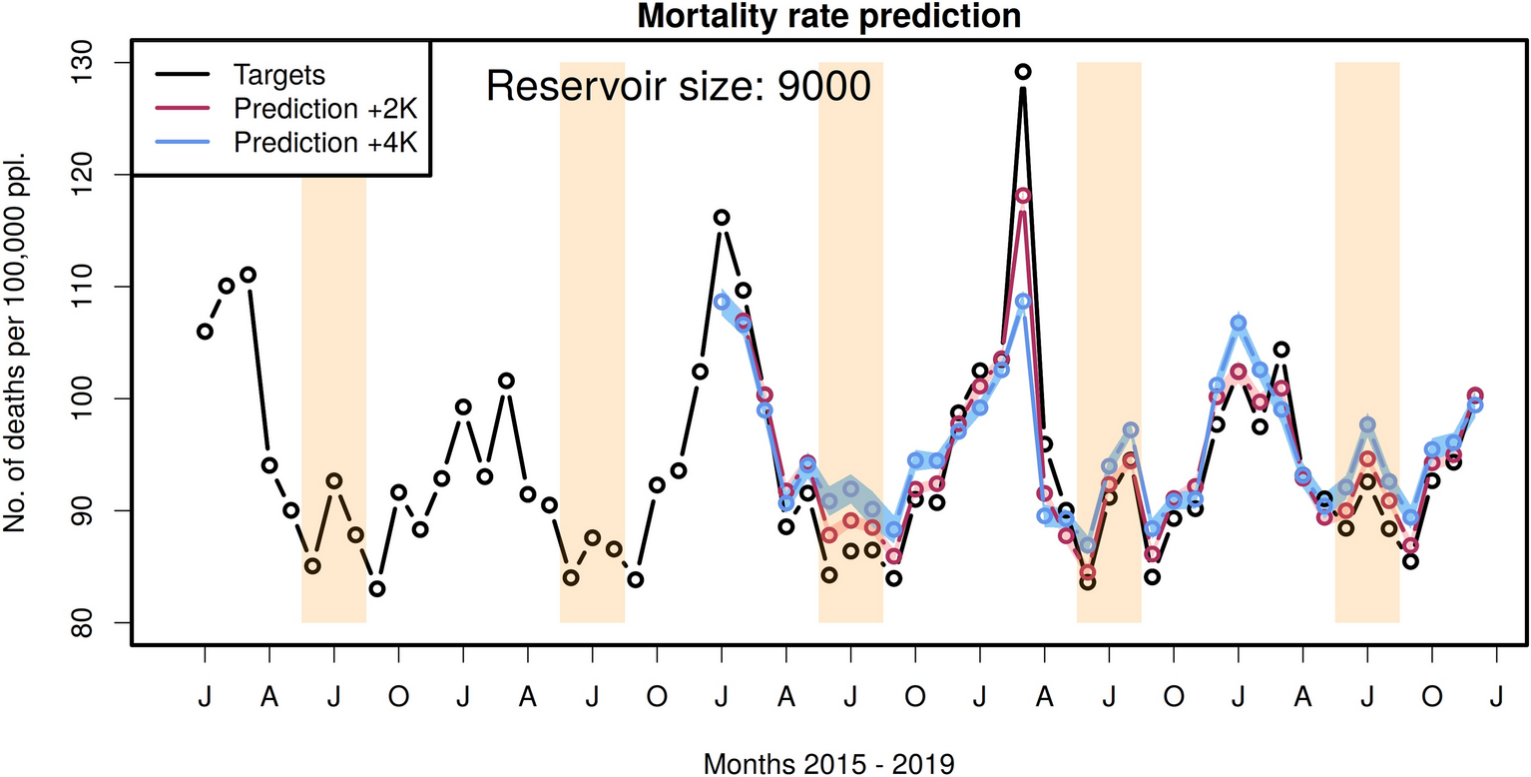
Mortality rate predictions for warmer climate storyline scenarios. Here, we show the prediction of the mortality rate for the warmer world scenarios: +2K in red and +4K in blue, along with the target values of the recent climate scenario on which the network was trained (black). The shaded areas represent one standard deviation from the ensemble mean values. The periods highlighted with orange background are the summer months (June–August). The warmer world scenarios are available only from 2017.

In Fig. 5, we show the mortality rate prediction for the two climate storylines investigated in this study. We calculate ensembles of trained ESNs and predictions to test the robustness of the approach since the initialization of the ESN’s input layer and reservoir are performed with random numbers. The ensemble size is 25. The blue and red shaded areas indicate the ensemble spread, i.e. the mean ± one standard deviation (SD), while the circles represent the mean values for each month. The SD for the +2K runs ranges between 0.42 and 1.0, for the +4K runs between 0.64 and 1.32, respectively. This is in the range of 0.5 to 1% of the target values, showing that the results are robust.

**Fig. 6:**
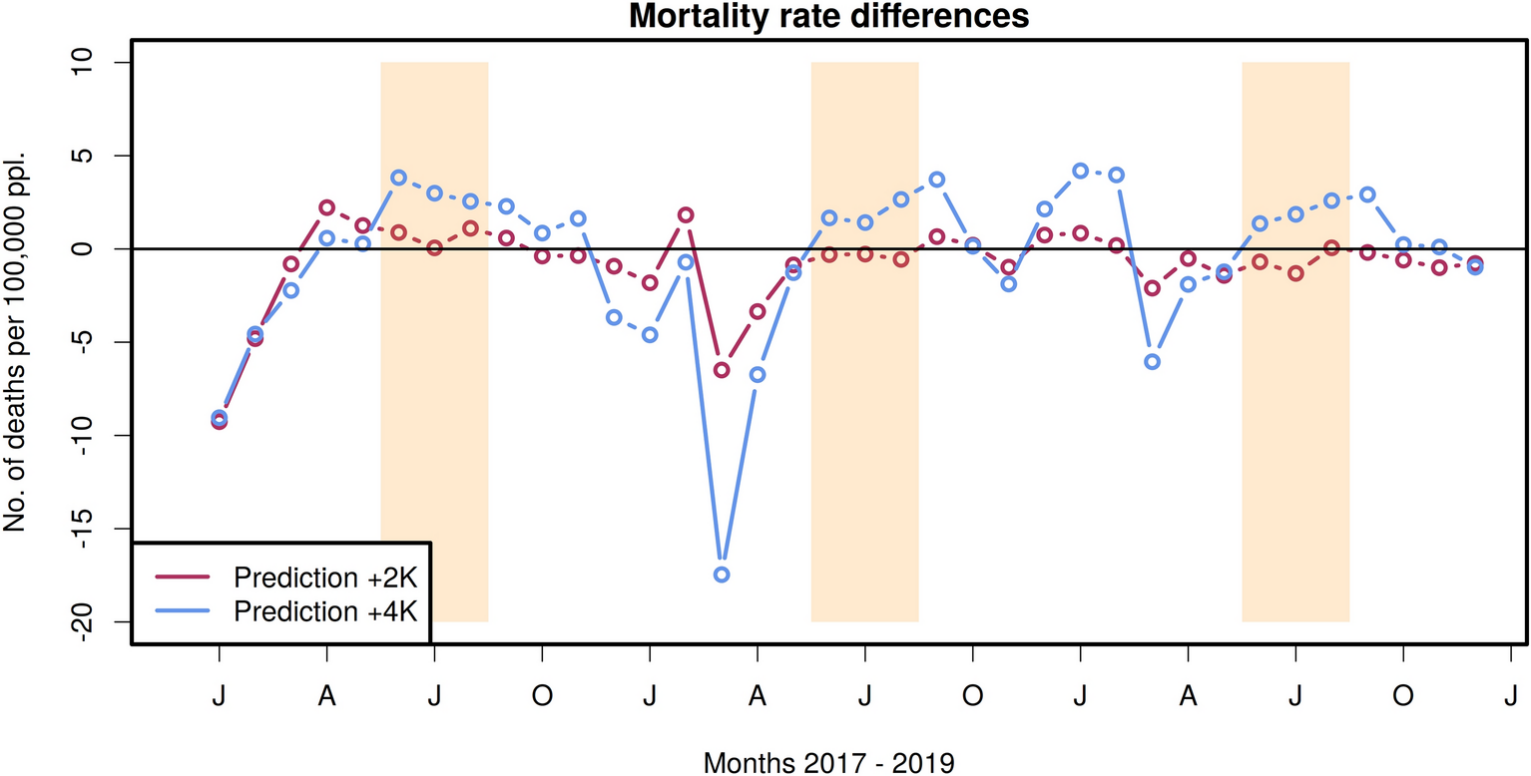
Differences between predictions and current mortality rates. Shown are the +2K scenario (red) and +4K scenario (blue). The intervals with the orange highlighted background are the summer months (June–August).

The difference between the predictions for the +2K and +4K scenarios and the target values representing the current mortality rates is shown in Fig. 6. During the summer months, the values are elevated while in winter the values are lower than in the reference period. The increase in mortality rate in the summer months is negligible for +2K storyline and 4-5 per 100,000 people for the +4K storyline. The latter corresponds to about 3,200–4,000 additional fatalities in Germany per month.

## Discussion

The storyline simulations from which input data for the ESN training are taken where computed for certain global CO2 levels. These CO2 levels correspond to mean global temperature increase of +2K and +4K w.r.t. pre-industrial time. The temperature increase may vary for different regions. However, storyline simulations are used investigate how extreme events enfold under climate conditions that represent certain global warming levels. In that sense the results obtained here also correspond to the global warming levels while regional temperature increase for heat waves can be much higher than on average (cf.^34^ ). This is also illustrated in Fig. 3 for four different locations in Germany.

The predictions of mortality rates in warmer world scenarios show elevated values in summer months and decreased values in winter months. We interpret those results as higher mortality due to higher heat stress in summer. High day temperatures in combination with high temperatures at night are a major cause of fatality^38^.

A decrease of mortality in winter time is plausible since warmer winters may lead to less severe influenza waves. However, this is hard to quantify since the impact of a influenza wave also depends on many other factors such as vaccination rate, effectiveness of the vaccination, etc. Therefore, this study’s analysis is focused on the summer months and heat-related mortality. Even with heat related mortality there are factors other than the ambient temperature. After the 2003 heatwave in Europe with thousands of fatalities among elderly people as reported by^39^ preventive measures have been taken in many European countries. This has lead to a reduced number of fatalities in the severe 2006 heatwave in comparison to the previous heatwaves without those measures^40^. However, ^21^ show that despite the evident adaption to heat, high temperature periods in summer still pose a significant thread to human health in Germany.

In order to reduce computational costs we used only monthly maximum temperatures as input data. However, the results show that our network is able to model the connection between temperature inputs and mortality on a very accurate level. The spatial extend of input data is significantly larger than the investigated domain. Spatially extended high temperature distributions allow the conclusion that the temporal extend at one location in or near the center is also high since the weather conditions would not change very quickly if high temperatures are observed all over the domain. Also, in the literature the influence of minimum temperature on cardiovascular diseases and mortality has been shown to be important^41,42^. We have run experiments using maximum values of daily minimum temperatures per month as additional input. This did not further improve the results for Germany. Therefore, the use of monthly maximum temperatures is a reasonable choice. Furthermore, humidity does not play a significant role for our results. We ran experiments using the maximum web-bulb temperature but the network training was not improved. However, should the method be applied to other regions where either the night temperatures or wet-bulb temperature is of more importance, it is straightforward to include them as input data for the ESN.

Another interesting question is how results would differ for different subregions of Germany. However, this is out of the scope of the state-of-the-art climate simulations (CMIP6 model, with horizontal resolution of approximately 100 km), whose resolution is still too low and does not allow that kind of investigation. Nevertheless, our study can be a first step in this area of knowledge that can be updated when the CMIP7 models are available.

Taking those facts into consideration, this analysis gives a hint as to what the impact of future climate change concerning mortality could be. Society and politics have to take the appropriate measures not only to prevent the warming as far as possible but also to reduce the risk of death under such climate conditions should they occur.

One drawback of ESN is that techniques that attribute the outcome to specific (parts of) the input data such as layer-wise relevance propagation cannot be used due to the cyclic nature of the network. However, the advantage of yielding robust results with short input data time series outweighs that disadvantage of lack of interpretability.

## Data Availability

The target mortality and population data used in this study are publicly available on the website of the German statistical service (see reference numbers in “Data” section of the main text). The climate model outputs used as input data for the network training and mortality prediction are available at https://zenodo.org/record/8014199. The R code used in this project is available from the authors upon request. Please contact R. Schachtschneider (reyko.schachtschneider@gfz.de) for further information.
